# Families' Perceptions of Powered Mobility for Participation in Children With Spinal Muscular Atrophy Type 1: A Photovoice Study

**DOI:** 10.1111/hex.70278

**Published:** 2025-05-07

**Authors:** María Coello‐Villalón, Cristina I. Díaz‐López, Purificación López‐Muñoz, Helena Romay‐Barrero, Soraya Pacheco‐da‐Costa, María Plasencia‐Robledo, Egmar Longo, Rocío Palomo‐Carrión

**Affiliations:** ^1^ Department of Nursing, Physiotherapy and Occupational Therapy, Faculty of Physiotherapy and Nursing Universidad de Castilla‐La Mancha Toledo Spain; ^2^ Research Group of Pediatric and Neurologic Physiotherapy, IMPROVELAB Toledo Spain; ^3^ Asociación de Padres de Niños con Dificultades en el Desarrollo—APANDID Toledo Spain; ^4^ Neuromusculoskeletal Physical Therapy in Stages of Life Research Group (FINEMEV), Physical Therapy Degree, Department of Nursing and Physical Therapy, Faculty of Medicine and Health Sciences Universidad de Alcalá Madrid Spain; ^5^ Centro de Atención Temprana El Grao, Hermanas Hospitalarias Valencia Spain; ^6^ Department of Physical Therapy Federal University of Paraiba João Pessoa Brazil

**Keywords:** barriers, children, emotional well‐being, participation, photovoice, powered mobility, spinal muscular atrophy

## Abstract

**Background and Purpose:**

Spinal muscular atrophy type 1 (SMA1) is a neuromuscular disorder that severely limits movement and autonomy in young children. Early powered mobility has proved to be a valuable intervention to promote participation, social engagement and emotional well‐being. To understand the potential impact of powered mobility, it is critical to explore children and families' experiences with participatory methodologies, such as the photovoice method.

**Objectives:**

To explore families' perceptions of powered mobility for participation in children with SMA Type 1, with a focus on emotional well‐being, social engagement and accessibility.

**Methodology:**

A qualitative descriptive study using the photovoice methodology was implemented, in which families documented their perceptions and experiences through photographs and reflective narratives over a period of 4 weeks. Children were encouraged to use a power mobility device in different activities in natural environments, after a training intervention of 12 weeks with power mobility. Families were asked to capture their experiences through photos and participate in different interviews to report their perceptions. Data was collected through photos documentation and during interviews.

**Setting and Participants:**

This study was conducted in natural environments, home and community, in Spain. The participants were six children with SMA1 (aged 13–28 months) and their families.

**Key Findings:**

Three major themes emerged: (1) Emotional and Social Engagement: Families reported that powered mobility enhanced children's confidence, emotional expression and ability to interact with family members and peers. (2) Barriers to Accessibility: Families encountered challenges such as limited home space, restricted public accessibility and the need for individualised adaptations. (3) Collaboration as a Key Factor: Parents emphasised the importance of collaboration with professionals in facilitating meaningful use of powered mobility.

**Interpretation:**

The findings align with previous research demonstrating the benefits of powered mobility in promoting independence, social interaction and participation. However, this study also highlights persistent environmental barriers that continue to limit full social inclusion. Addressing these challenges is crucial to maximising mobility‐related gains.

**Conclusions and Implications:**

This study highlights the importance of addressing accessibility barriers and promoting interdisciplinary collaboration to maximise the benefits of powered mobility for children with SMA1. Incorporating participatory methodologies such as photovoice provides a powerful means for families to voice their experiences and advocate for inclusive mobility solutions.

**Patient or Public Contribution:**

Families were actively involved in multiple stages of the study, including its design, data collection and interpretation and dissemination of the results. Using the photovoice method, parents documented their children's experiences with powered mobility through photographs and comments, highlighting both benefits and challenges. Their input was crucial in identifying real‐life barriers and needs, ensuring that the study reflected the authentic experiences of families navigating powered mobility. Additionally, they provided valuable insights during interviews, contributing to a deeper understanding of the emotional, social and practical impacts of the intervention. Their perspectives helped to shape the analysis and reinforce the importance of personalised mobility solutions.

## 1 Introduction

Spinal muscular atrophy (SMA) is characterised by degeneration of the alpha motor neurons of the anterior horn cells of the spinal cord, leading to progressive proximal muscle weakness, hypotonia, areflexia and atrophy. It has an estimated incidence of 1 in 11,000 live births [1], and it is usually classified into four different types [1, 2]. SMA type 1 (SMA1), also called Werdnig–Hoffmann disease, presents symptom onset within the first 6 months of life and may lead to severe limitations in functional mobility and participation. Despite not having cognitive issues, children with SMA1 may have delays in the acquisition of some skills because of the limitations mentioned, which cause delays in motor milestones with mobility restrictions and great difficulties in achieving independent sitting or walking [3, 4].

Mobility plays a crucial role in child development, providing the means to explore, interact with peers, form friendships and become part of a group across different environments. Independent mobility facilitates the acquisition of other developmental skills, including language and cognitive abilities [5]. Without this autonomy, children face restrictions in exploring their environment, learning and participating in natural settings. For children with SMA1, these limitations underscore the importance of early interventions, such as powered mobility, to foster independence and inclusion [6].

However, the traditional concept of early powered mobility can be misleading. As highlighted by Sabet in their ON Time Mobility framework, so‐called ‘early’ powered mobility is often provided later than optimal. This framework asserts that all children have the right to be mobile at every stage of their development, emphasising key principles such as timing, urgency, multimodal access, frequency and sociability. From this perspective, powered mobility should not be viewed solely as a compensatory tool but rather as a fundamental right that enables children to explore, interact and develop autonomy within their communities.

In this way, early powered mobility not only supports independence and peer socialisation but also enhances participation in family and community activities. As self‐initiated locomotion is crucial for cognitive, perceptual and social development, introducing powered mobility early in the lives of children with SMA1 is essential for improving their functional abilities, participation and quality of life [7, 8]. Despite its benefits, powered mobility is often viewed narrowly as an individual ‘fix’ or compensation for mobility challenges rather than a vital tool for fostering access, participation and inclusion in broader social and physical spaces [9]. To truly understand its potential impact, it is critical to explore the lived experiences of children and their families.

Participatory methodologies, such as the photovoice method [10], offer an effective methodology for capturing these perspectives. Photovoice, guided by participatory action research (PAR) principles [11], empowers participants to document their experiences through photography, enabling them to articulate their challenges, needs and aspirations in a creative and accessible manner [12]. This methodology is particularly impactful for children with disabilities, as it offers a means that transcends verbal or literacy limitations. By documenting their environment and use of powered mobility, children and their families can share their unique perspectives, revealing barriers and opportunities for enhancing mobility and participation [6]. The use of photovoice not only fosters community engagement but also enhances participants' self‐efficacy and raises awareness of local resources, promoting social transformation [13]. For children with SMA1, this method is particularly relevant, as it enables families to take photographs at their convenience without the constant presence of researchers. These images serve as powerful tools to represent the interests and needs of both the child and their family, offering insight into the barriers to mobility and participation in their natural environments [14]. In the context of children with SMA1, the use of the photovoice method is particularly relevant, as it allows for the capture of photographs of the children's environment and their use of powered mobility, providing a platform to represent their needs and challenges. The images captured provide a direct view of the barriers they face and the areas where intervention may be necessary [15]. Although the use of photovoice as a tool to describe experiences from the perspective of children and their families regarding the implementation of powered mobility interventions remains limited, it is crucial to recognise that the voices of families are central to this process [16]. Families are the key players [17], as they interact with powered mobility on a daily basis and experience firsthand the advantages and challenges they bring. The use of photovoice not only allows children and families to document their experiences but also provides them with a platform to share their perspectives on the barriers they face in daily life and the improvements that could be made to enhance their participation in family and community settings [18].

Therefore, the aim of this study was to describe the experiences of children with SMA1 and their families with an early powered mobility intervention. The study also sought to explore how these interventions influence participation in everyday activities and interactions within their natural environments, using participatory methods such as photovoice to capture their perspectives.

## 2 Methods

### 2.1 Study Design

A qualitative participatory narrative study was carried out within the randomised clinical trial (RCT) ‘*AMESobreRuedas’* [19]. The overall intervention of the RCT lasted 16 weeks and consisted of a structured training programme in which children with SMA1 had three 30‐min‐power mobility sessions per week, during 12 weeks, and a follow‐up period of 4 weeks of free use of the power mobility device. Children used a modified ride‐on car (MROC) individually adapted in their natural environment, for home and community activities. During the first 12 weeks, the sessions were supervised by pediatric physiotherapists, with active family involvement to enhance mobility and social interaction. For the follow‐up period, from Week 13 to Week 16, families were asked to encourage their children to keep on using the MROC during different activities, without having the pressure of adhering to the structured weekly protocol. During this follow‐up period, with unrestricted use of the MROC, families were invited to participate in the photovoice methodology, documenting their experiences with powered mobility.

### 2.2 Ethical Approval

The study complies with the Declaration of Helsinki for ethical principles [20], as well as with the Spanish Law on Personal Data Protection and Guarantee of Digital Rights, from December 2018 [21], which oversees research involving individuals with disabilities to ensure ethical compliance and participant safety. The RCT ‘*AMESobreRuedas’* [19] was approved by the Ethics Committee of the University Hospital in Toledo (Ref No. 842), and it is registered at www.clinicaltrials.org with the ID: NCT05589987.

Informed consent was obtained from the children's families for their participation in the RCT ‘*AMESobreRuedas’* [19], ensuring the confidentiality of all the data collected via a specific coding system to refer to each child and family. In addition, explicit consent is obtained from each family for the capture and disclosure of images. Pseudonyms were used to protect the privacy of all participants.

### 2.3 Patient Involvement

This study was based on PAR principles [11] and public and patient involvement (PPI) methodology [22, 23], ensuring that families were actively engaged in all stages of the research process. Since the children were between 13 and 28 months old, obtaining direct verbal data from them was not feasible. Therefore, their experiences were interpreted based on observed behaviours, facial expressions and levels of interaction with their environment, alongside their families' perceptions of these experiences.

Families were invited to document their experiences through photographs and descriptive narratives that reflected their responses on emotional well‐being and social engagement and challenges of accessibility they found at home and in the community. The families subsequently selected the most meaningful photos and shared their interpretations, along with descriptive narratives, with the research team.

Children and families were further involved in data interpretation and participated in collaborative reflection sessions to validate themes and findings, ensuring that the outcomes accurately represented their lived experiences. While the dissemination strategy is still being developed, plans include sharing results with families, healthcare professionals and the broader community through public presentations and publications. This collaborative methodology underscores the commitment to empowering participants and amplifying their voices in the research process. To enhance the credibility and trustworthiness of the findings, collaborative reflection sessions were conducted following the final data collection phase. These sessions allowed families to validate emerging themes, refine interpretations and co‐construct meaning from their experiences alongside the research team.

Families were also encouraged to disseminate the findings to other families, healthcare professionals and the broader community through tailored public presentations and knowledge translation initiatives.

### 2.4 Participants

Families of children diagnosed with SMA1 who were willing and able to participate in the narrative and photographic parts were included in the RCT ‘*AMESobreRuedas’* [19]. The exclusion criteria were as follows: families with significant language or communication difficulties that prevented effective engagement with the research team and/or any factors that could pose ethical concerns or safety risks to the child or family during the study.

### 2.5 Study Procedure

**Figure 1:**
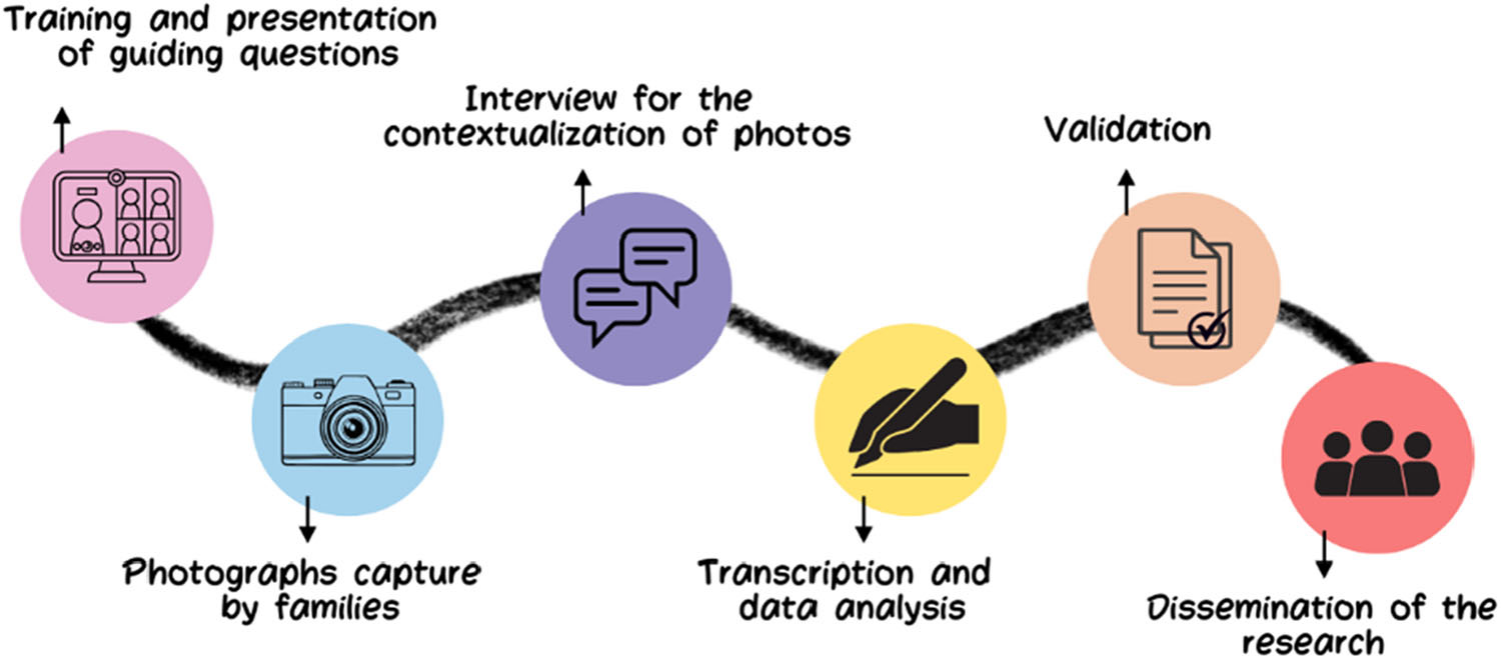
Stages to follow for planning and obtaining photographic results.

Figure 1 provides a structured flow chart of the full process, from the training phase to the final dissemination of the research, sharing the results. It highlights key stages such as guiding question training, photographic documentation, contextualisation interviews, thematic transcription, validation sessions and community dissemination. This representation ensures clarity on how families' narratives were gathered, analysed and integrated into the study's findings.

#### 2.5.1 Introduction to Photovoice

Before starting the study, all families that participated in the RCT ‘*AMESobreRuedas’* [19] were invited to a face‐to‐face session conducted by the research team to provide comprehensive training that was divided into two parts.

In the first part, all families were introduced to the photovoice methodology [24], and the main aims of the study were discussed. This session included an explanation of the importance of capturing experiences visually and of the guidelines on how to take meaningful photographs. The second part of the training was practical. Families were presented with five questions related to their daily lives, outside of powered mobility, to familiarise them with the method and facilitate their understanding. These questions included: (1) What activities do you enjoy most during your day? (2) How do you spend time with your family? (3) What challenges do you face in your daily life when you are not using the powered mobility device? (4) How do you feel emotionally during your daily activities, such as when you interact with family or participate in activities outside of mobility? (5) How do you describe your daily routine when you are at home or out in the community?

The questions were designed to help families reflect on their daily experiences and understand how to document them visually. The training allowed participants to familiarise themselves with the photovoice process, making it easier for them to apply the method to capture meaningful moments and experience. During this session, the participants were provided with materials, such as cameras or smartphones, and were taught how to use them effectively to document their experiences independently from home.

##### 2.5.1.1 Photographic Data Collection

This session was designed to provide a comprehensive understanding of the photovoice methodology [10] and its role within the PAR framework [11] and PPI principles [22].

**Figure 2:**
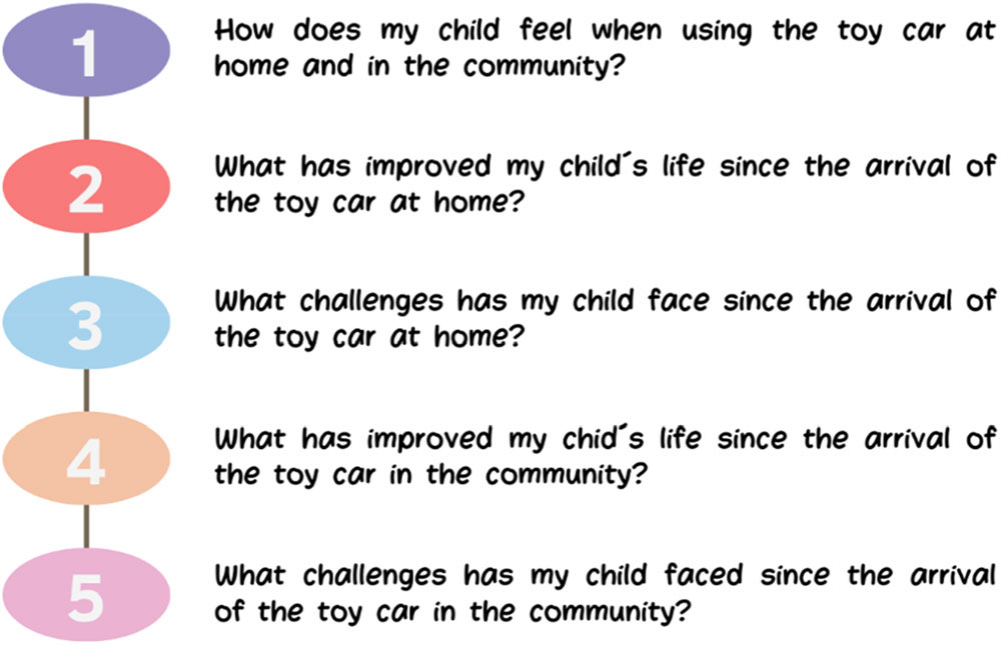
Guiding questions for reflective photography.

During this session, families received guidance on how to capture high‐quality photographs that reflect their children's experiences while using powered mobility. A structured script with guiding questions (Figure 2) was shared to help families focus on capturing meaningful moments.

##### 2.5.1.2 Narrative Collection

**Figure 3:**
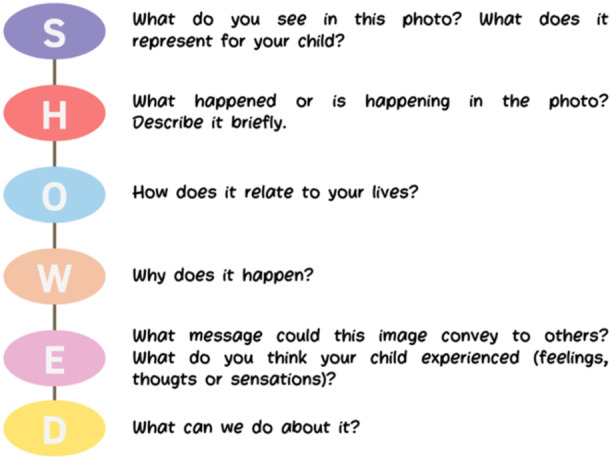
Semi‐structured script based on the SHOWeD methodology adapted to guide photographic reflection.

Families were asked to capture 4–5 photos per week during the final 4 weeks of the study (Weeks 13–16), resulting in a total of 16–20 photos per family. These photos documented children's daily routines, interactions with family and peers, and mobility challenges or successes. After this period, a discussion group meeting was held between the families and the research group, which was based on the adapted version of the SHOWeD methodology (Figure 3) [10]; families reflected on selected photographs and thematic findings to discuss the content of the photographs and explore the experiences of participation during the powered mobility intervention with the MROC. The process involved reviewing the photographs, answering the guiding questions and aligning the findings with their lived experiences. To ensure an equal voice in the interpretation of the photos and avoid ‘group think’, each family was invited to share their individual interpretations of the photos before any group discussion took place. This approach ensured that all perspectives were heard and valued. The discussion was then facilitated to encourage open dialogue while preventing the domination of any single viewpoint, ensuring that each family had an equal role in interpreting the photographs.

Figure 3 provides a structured flow chart of the full process of narrative collection, from the training phase to the final exhibition of findings. It highlights key stages such as guiding question training, photographic documentation, contextualisation interviews, thematic transcription, validation sessions and community dissemination. This representation ensures clarity on how families' narratives were gathered, analysed and integrated into the study's findings.

At the end of the study, a systematic analysis was conducted by integrating the narratives and photographs to identify significant and recurring themes from the families' experiences. This process involved transcribing the discussions, categorising data collaboratively between families and researchers, and validating the findings. These steps provided a comprehensive understanding of how the powered mobility intervention influenced the participation, social interactions and quality of life of children with SMA1 and their families, culminating in a community‐focused presentation to share insights and promote awareness.

All stages of the process that required interaction with the research team—such as training, interviews for contextualisation of photos, transcription, data analysis and validation—were carried out remotely via Microsoft Teams. The families were able to upload their photos independently, outside of the app, and participate in the process at their convenience. The stages of this process are outlined in Figure 1.

###### 2.5.1.2.1 Data Analysis

For the analysis of the data, feasibility results were reported qualitatively [10, 25]. We conducted a thematic analysis approach based on Braun and Clarke's framework [26], supported by ATLAS.ti software to facilitate coding and theme development. The process followed these steps:

– Data Transcription and Initial Coding: All audio‐visual data from family interviews and reflection sessions were transcribed verbatim. Two researchers (D.L.‐C. and P.D.‐S.) analysed the data independently, followed by a discussion among the research team to discuss the preliminary results.
– Thematic identification: Codes were clustered into preliminary themes, which were refined through team discussions and validated by families during collaborative reflection sessions to ensure accuracy.
– Final Themes: The final thematic structure, developed through iterative coding and validation, is presented in the ‘Results’ section.

####### 2.5.1.2.1.1 Rigour

To ensure the trustworthiness of the study [27, 28], we followed established criteria for credibility, transferability, dependability and confirmability:

– Credibility: Data and researcher triangulation were ensured using semi‐structured interviews and field notes, with two independent researchers analysing the data.
– Transferability: Detailed descriptions of the study design, participant characteristics, sampling strategies and data collection and analysis procedures allow for application in similar contexts.
– Dependability: An external researcher conducted an independent audit of the study's research protocol, focusing on the methods and study design to ensure consistency and reliability.
– Confirmability: Triangulation and reflective reporting minimised bias and increased objectivity.

## 3 Results

During the 4‐week follow‐up period of the RCT ‘*AMESobreRuedas’* [19], six families participated in the study documenting their children's experiences with the MROC. Although not all families were Spanish, all the children were born in Spain and had a good understanding of the Spanish language. Table 1 presents the characteristics of the children and their families.

**Table 1:** Summary of child and family characteristics.

ID	Age (month)	Therapy support	Wheelchair user	Attends school	Parent's age (years)	Family structure	Parents' education level
Child A	13	Yes	No	No	38	Twin siblings and parents	Graduates
Child B	15	Yes	No	No	33	Only child and parents	Secondary education
Child C	23	Yes	No	No	25	One sister (5 years old) and parents	Secondary education
Child D	14	Yes	No	No	45	Parents	Graduates
Child E	24	Yes	No	No	29	Parents	Graduates
Child F	28	Yes	Yes (passive)	No	33	One sister (10 years old) and parents	Secondary education

The research team received 106 photographs. In individual interviews, families selected 20 images that best represented their experiences.

### 3.1 Narrative Results

Thematic analysis revealed four primary themes: Emotions Experienced, Improvements at Home, Challenges and Barriers. These themes emerged from the qualitative coding of participant narratives, validated through collaborative reflection sessions. Below, we present each theme with illustrative examples from families' and children's experiences.

### 3.2 Well‐Being Emotions

Thematic analysis identified confidence, security and enjoyment as the primary emotional responses to the MROC. Since the children were unable to express their emotions verbally, their experiences were inferred from their body language, facial expressions and reactions to using the device, as well as from their families' accounts of these experiences. These emotional responses were therefore interpreted through the lens of parental observation rather than direct verbal communication from the children.

Confidence and autonomy: Families reported increased engagement over time. One mother noted, ‘Initially, my child was hesitant, but now he moves confidently across the room.’

Initial hesitation: Some children initially expressed fear. Child B's mother stated, ‘At first, he cried when placed in the car, but after a few sessions, he smiled and moved on his own.’

Parental observations: B's mother stated, ‘The changes have been drastic. Now, B happily moves wherever he wants. Although he still gets easily distracted, my son enjoys the MROC and the freedom of movement.’

Families described a range of emotional responses in their children during the initial stages of the powered mobility intervention, including expressions of curiosity, hesitation, excitement and joy.

This observation suggests that powered mobility had an impact on the child's emotional perception, although their experience was interpreted based on nonverbal cues.

Nevertheless, some families faced some challenging comments: A's mother stated, ‘*I believe people do not realize the pain that words can cause because if they do, they would think before speaking*.’

**Figure 4:**
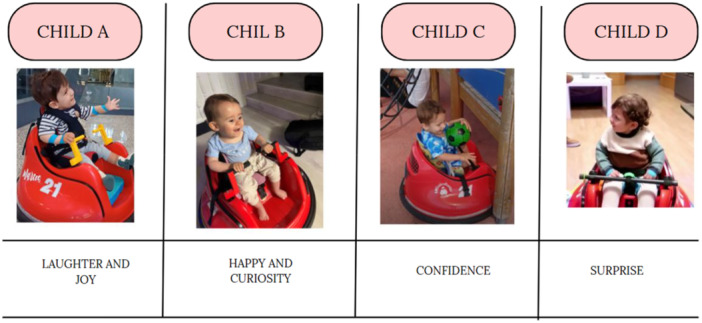
Emotions evoke by the modified ride‐on car.

Figure 4 shows some of the images selected by the family and the reported emotions the car produced in them.

### 3.3 Social Interaction at Home

Here, we highlight the responses of the two families.

– Child A's participation evolved throughout the training sessions; initially, his motivation was to chase and ‘run over’ his brother with the MROC. His mother laughed and commented, ‘*My son is used to his brother being faster and taking his toys, and now it appears to be he wants to get even. I love seeing them play together*.’ As the weeks went by, the environment changed, as did the people accompanying him and those he could interact with, allowing him to make more decisions.
– Child C was able to interact with all the family members, including the pet, and looked happier. The mother's remark, laughing, stands out: ‘*My child's first motivation with the car toy was to interact with the dog, not with me or his father*,’ acknowledging the happiness that the experience brought to everyone at home.

**Figure 5:**
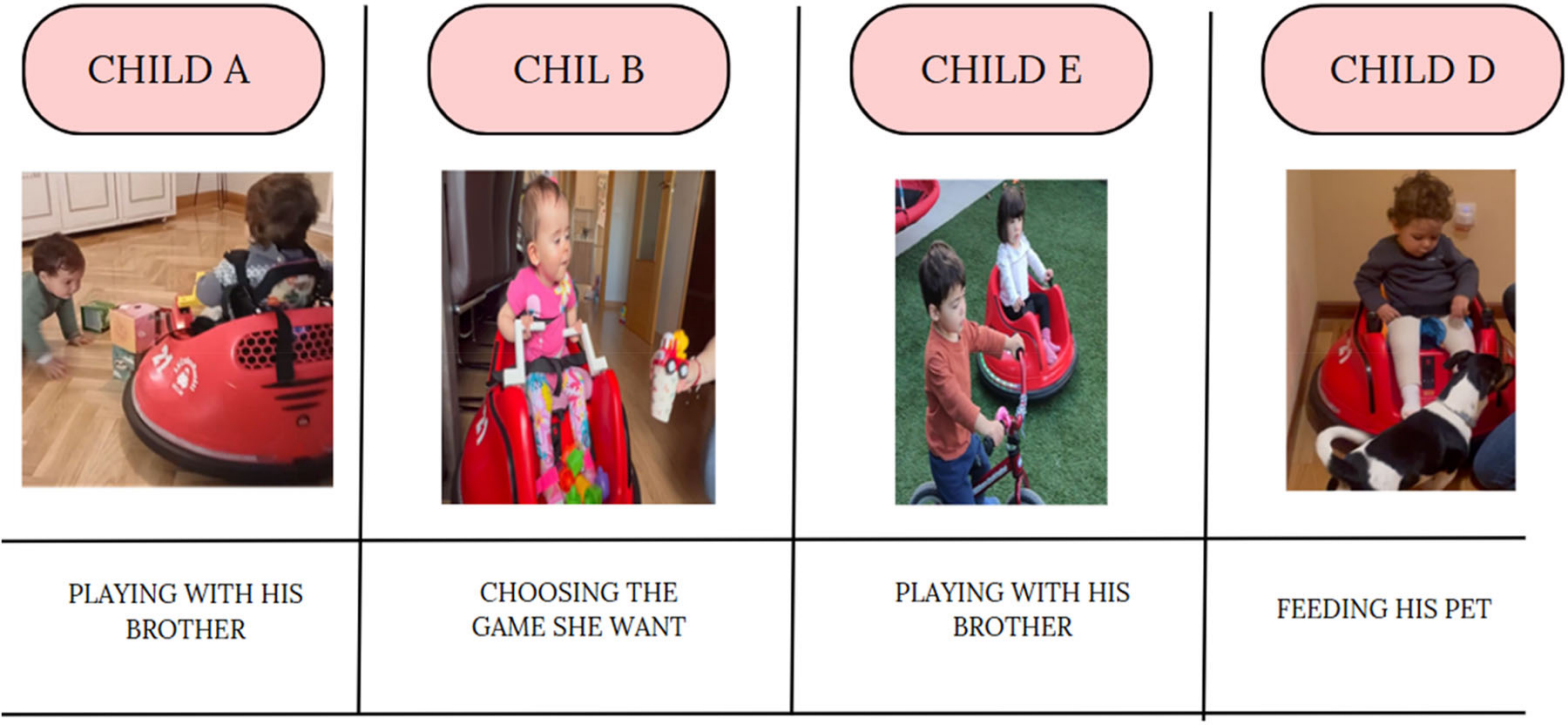
Improvements observed after using the modified ride‐on car at home.

Figure 5 shows the improvements observed in children A, B, E and D after using the MROC at home, highlighting sibling play, decision‐making power when choosing toys, and feeding the pet.

### 3.4 Social Interaction at the Community

Families reported that, beyond the home setting, children had opportunities to engage in a variety of activities while using the MROC. These included going shopping at a supermarket or a mall, going to a park, going for a walk with the family, observing nature and actively approaching it, or feeding a horse.

Several families highlighted that the outdoor environment is more stimulating for them. One mother mentioned, ‘*Of all the benefits that the car toy could bring us, I never thought it would help him make friends. Before, just thinking about coming to the park was an effort; it scared me, but now I see him happy*’. Another mother commented, ‘*It was very beautiful to see him take the initiative outside the home for the first time. To capture his attention, how he enjoys watching the doors of the shopping center open and close and how he has a clear vision of what he’* wants, another family member reflected: ‘*Seeing her approach the animals with such confidence and joy, on her own, was deeply moving. It felt like she was truly engaging with the world around her.’*

**Figure 6:**
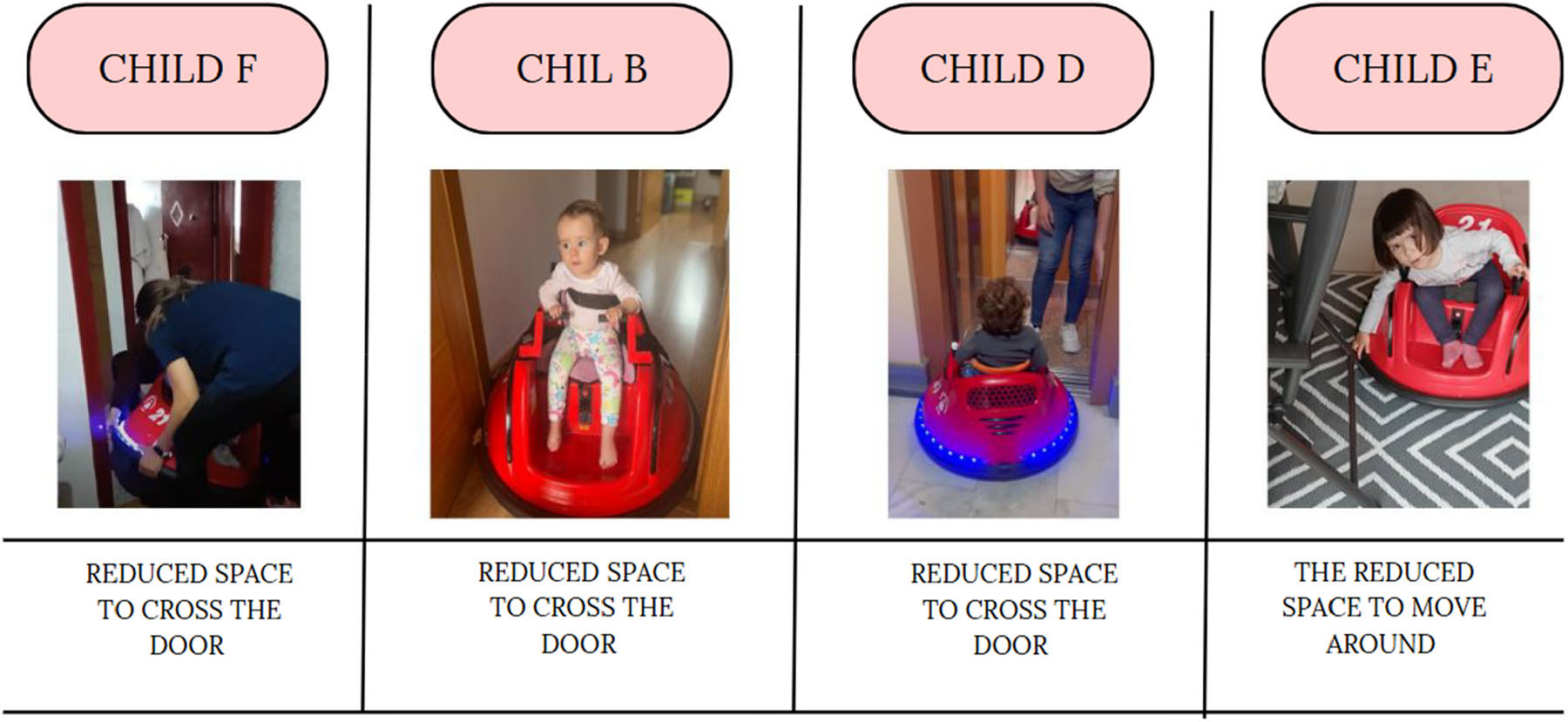
Difficulties of moving around the house with the modified ride‐on car.

Figure 6 shows the advantages provided by the MROC to children A, C, F and D in the community. These advantages include allowing them to move around the shopping mall, play in the park, independently travel to equine therapy and enjoy nature by observing plants and everything that catches their attention.

### 3.5 Accessibility Challenges

Families reported some challenges while using the MROC.

On the one hand, physical limitations at home were faced: Child F's mother described difficulty navigating door‐frames, stating, ‘She gets frustrated when the car doesn't fit through the doorways.’

**Figure 7:**
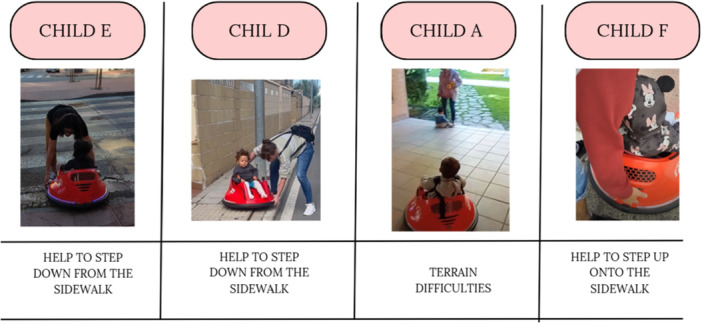
Difficulties and limitations encountered while using modified ride‐on cars in the community.

Figure 7 shows the four images selected by the parents that best represent the difficulties of moving around the house with the MROC, such as the reduction in space and the inability to cross the door‐frame.

Families also reported physical and interaction barriers and limitations in the community, such as the difficulty of getting onto sidewalks or uneven terrain, the narrowness of sidewalks and the general lack of adapted access in the outdoor environment.

Child E's father said, ‘There are no ramps in some areas, making it impossible to use the car outdoors.’

**Figure 8:**
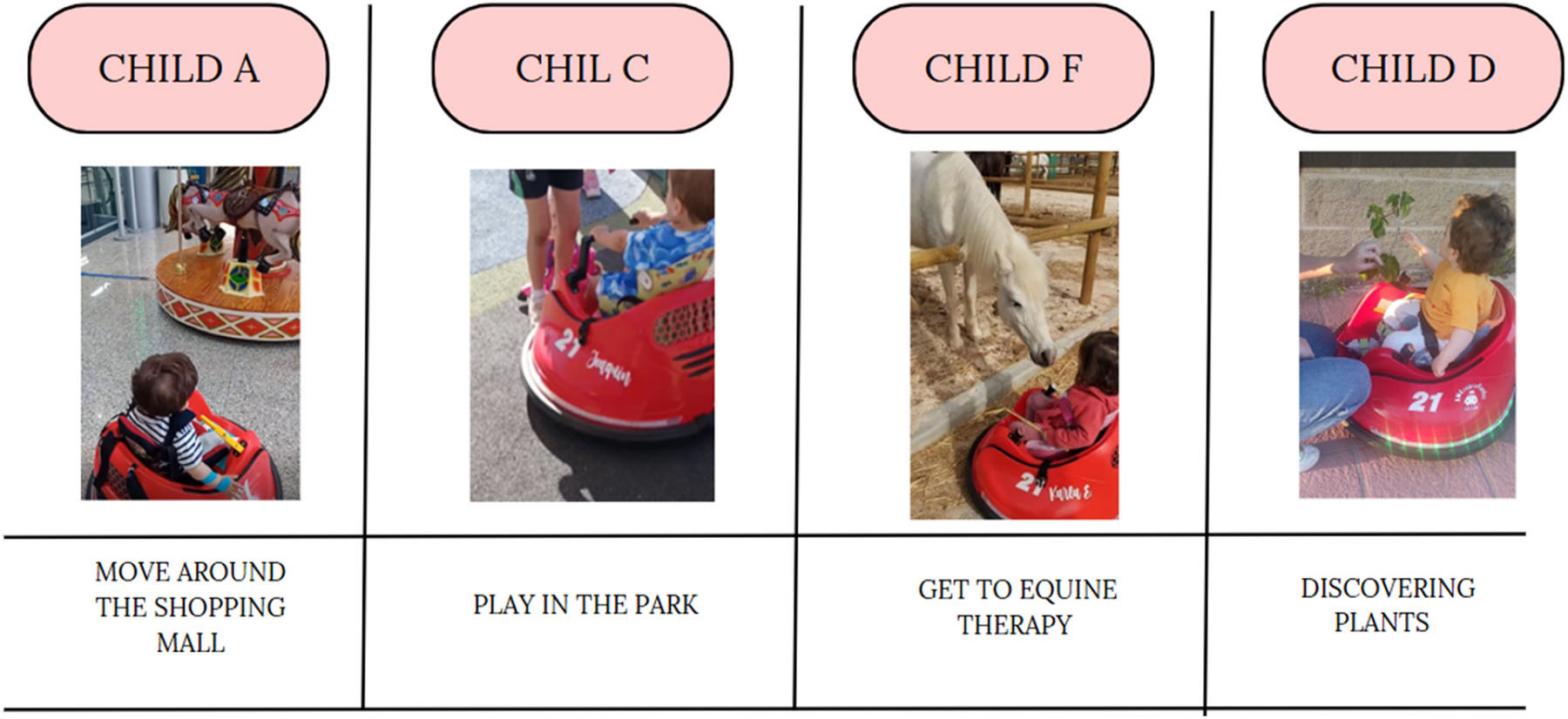
Advantages provided by the modified ride‐on car in the community.

Figure 8 shows the difficulties and limitations encountered by children E, D, A and F while using the MROC in the community.

## 4 Results of the Collaborative Reflection Sessions

### 4.1 Common Themes Identified

Thematic analysis identified four main themes related to the powered mobility experience: Impact on Family Dynamics, Daily Life and Play, Limitations and Barriers, and Independence. These themes emerged from family narratives and were validated through collaborative reflection sessions.

– Impact on Family Dynamics: Families reported that recording their children's interactions during training sessions was sometimes challenging, particularly when children were tired or less engaged. However, they described feeling more confident over time in documenting experiences.
– Daily Life, Play and Participation: Families shared how the MROC had positively influenced simple daily routines, enriched solitary playtime, explored corners of the house and fostered participation in family activities with siblings and pets. It also allowed going out, such as going to therapy, the supermarket or family walks.
– Limitations and barriers: Despite positive outcomes, ongoing challenges are acknowledged in three main areas: (i) Community challenges, particularly those concerning accessibility in certain public spaces. The participants highlighted that many environments remain inadequately adapted for individuals with reduced mobility, limiting their ability to explore freely. (ii) Home challenges: These challenges include the lack of space to move around, the inability to cross doors and the need to rearrange furniture to clear an area for practice. (iii) MROC limitations: Families highlighted the effectiveness of powered mobility interventions in increasing independence, but some people pointed to mechanical issues or design limitations that sometimes hampered their child's experience.
– Independence: Families have shared photographs and anecdotes that highlight the importance of giving the opportunity to decide and choose. Parents reported that children actively navigated their surroundings, approached specific toys or people, and displayed excitement when engaging with their environment.

Some comments made by parents concerning the different common themes identified are shown in Table 2.

**Table 2:** Comments made by families of the different common themes identified.

Impact on family dynamics	‘If we hadn't had the constant support of their therapists, I think we would have done far fewer training sessions. However, now that I see the results, I would say that I would do it all over again’
	‘The recordings were like a roller coaster; at first, he would get angry and cry because he did not want to sit in the car, then he would cry because he did not want to get out’
	‘Knowing we had many weeks ahead of us was stressful, but the direct contact with you and seeing our child's enjoyment helped us relax’
	‘Depending on the day, we had to ask the grandparents to look after the older sibling so that my husband and I could record and focus on him’
Daily life, play and participation	‘The car has opened up a whole new world for our son, allowing him to participate more actively with his siblings’
	‘It is incredible to be aware that we are living moments we never thought possible’
	‘We love the independence it provides, but we have had to work through some technical issues’
	‘I love seeing him play behind his sister and watching them chase each other or go after the ball’
Limitations and barriers	‘It is disheartening to realize that while our child has gained independence at home, we still face barriers when we go out’
	‘First, it is clear that the car has given my son independence, but this independence depends on where we are, we need to think first about where to go, if the plan is compatible with the car’
	‘We love the independence it provides, but we have had to work through some technical issues’
	‘Sometimes playing with the car at home becomes very repetitive because we could only play in the living room and entrance. There was no option to do anything else, we could not fit’
Independence	‘I was surprised to see my son go straight to the cookie aisle when we entered the supermarket’
	‘When we arrived at the park, he went towards other kids who were playing ball’
	‘Seeing him enjoy small things like playing with the automatic doors at the shopping mall, going in and out by himself’
	‘Saying we're going for a walk and watching him go to his room to get a doll because he wants to take it with him’

## 5 Discussion

This study provides an enriching way to understand the experiences of families with children with SMA1 during a powered mobility intervention at home and in the community through images, comments and reflections. Since the children were too young to provide direct accounts, their experiences were interpreted through observed reactions, family testimonies and collaborative reflection sessions.

It was developed within the last 4 weeks of the RCT ‘*AMESobreRuedas’* [19] when children had unrestricted use of the powered mobility device, allowing them to engage with it freely in various activities. The decision to collect photos only during the final 4 weeks was intentional, balancing methodological and practical factors. This timing allowed participants to fully engage with the structured intervention during the first 12 weeks of the RCT *‘AMESobreRuedas’* [19], develop a deeper understanding of their experiences and reflect on changes in mobility, accessibility and independence before documenting them. Introducing photovoice too early could have shaped initial perceptions or disrupted the natural adaptation process. Delaying it ensured more authentic reflections while minimising stress during the structured intervention. Additionally, logistical factors, such as training participants and fostering comfort with photo‐based expression, influenced this choice.

The results indicate that powered mobility with the MROC led to significant changes in the children's emotional responses [10, 29], independence and participation [30, 31, 32, 33, 34], as well as the barriers and difficulties encountered during the intervention [35, 36, 37]. Our findings are in accordance with those of previous studies that highlighted the benefits of powered mobility for the participation, social skills and communication of children with disabilities [38, 39]. Similarly, studies using the photovoice method have explored the lived experiences of children and families using powered mobility. A study of children with congenital Zika syndrome [29] reported that powered mobility facilitated participation, increased mobility independence and improved family interactions while also highlighting environmental accessibility challenges. Additionally, research investigating the provision and early use of powered mobility through participatory methods [13] identified key themes related to mobility technology functionality, daily life participation, self‐advocacy and the complex interplay between families and industry. However, in contrast to studies that report successful integration of powered mobility into community settings, our research highlights persistent barriers related to public accessibility. These challenges often prevent full social inclusion, indicating a need for continued adaptations to improve accessibility. This contrast underscores the importance of anticipating these barriers to avoid frustration and ensure that powered mobility is fully integrated into children's lives [40].

One of the most prominent themes in our study was the impact of powered mobility on family dynamics. The intervention required active family involvement not only in the training sessions but also in decision‐making regarding mobility adaptations. Our findings suggest that families must be empowered to set realistic goals for powered mobility training [41]. Furthermore, the involvement of families not only in training sessions with the MROC but also in the design of the sessions—considering their daily routines—can reduce frustration and improve the training experience [42].

Children's lack of independent mobility is associated with reduced opportunities for playing, exploration and social interaction with peers and increases the risk for developmental, cognitive and psychosocial delays. In this study, powered mobility contributed to increased environmental exploration, goal achievement and interactions with siblings, ultimately fostering greater engagement in daily life [43]. The introduction of early powered mobility has been shown to facilitate learning, socialisation, self‐initiated movement and interactions, resulting in improvements in psychosocial and cognitive increases in curiosity and outgoing social interactions [44].

Despite these benefits, our study also identified significant challenges, particularly regarding barriers to mobility access and usability. As in other studies on powered mobility interventions [45, 46], issues related to device size and transport limitations were evident. However, our findings also emphasised that, beyond addressing these barriers, tailored adaptations to meet each child's specific needs are essential for facilitating independent exploration and social interaction. These adaptations are crucial for supporting developmental gains and promoting social inclusion [47].

The ability to explore and interact with the environment through powered mobility has a direct effect on children's social skills. As they gain more independence, they become more engaged in play and social activities, which, in turn, enhances their motivation, emotional well‐being and sense of agency [48]. Our study also underscores the importance of collaborative reflection sessions in refining thematic analysis. By involving families in the interpretation of findings, we ensured that their voices were central in shaping the study outcomes, reinforcing the participatory nature of the research.

### 5.1 Strengths and Limitations

One of the strengths of this study is the implementation of the photovoice method, which provided a creative and accessible way for families to document their experiences through images and narratives [10]. This methodology provides participants with space to express their feelings and offers insights into their experiences throughout the process. The thematic analysis of visual and textual data deepens the understanding of these experiences, highlighting the importance of continuous collaboration between professionals and families to ensure effective follow‐up. Given the significant impact of powered mobility on children's emotional, social and motor development, health professionals should integrate these devices into intervention programmes, tailoring adaptations to each child's specific needs. Additionally, policymakers should prioritise improving public accessibility to promote the social inclusion of individuals with reduced mobility.

The results highlight the importance of close and continuous collaboration between professionals and families to ensure proper follow‐up, personalised adaptations and positive experiences. This study underscores the opportunity to continue researching and developing innovative solutions that enable greater inclusion and participation of children with motor limitations in various settings, both at home and in the community.

Despite these significant findings, this study has several limitations. First, the small sample size—comprising only six children and their families—limits the generalisability of the results. Future studies with larger and more diverse samples could provide broader insights into the impact of powered mobility across different settings.

Second, although both parents and caregivers participated, the primary respondents in the study were mothers, which may introduce a maternal bias in the interpretation of findings. While the study aimed to incorporate diverse family perspectives, future research could explore how other caregivers, including fathers and extended family members, perceive the intervention.

Third, the reliance on parental reports and observations to infer children's experiences presents a methodological challenge. Although behavioural analysis and reflection sessions were used to mitigate this limitation, the lack of direct verbal accounts from children restricts a fully autonomous perspective. Future research should investigate alternative strategies to capture young children's experiences more directly, such as integrating nonverbal communication assessments or observational methodologies. Finally, accessibility remains a major challenge for powered mobility interventions. Despite advancements in adaptive mobility devices, physical and environmental barriers continue to limit children's full participation in community settings. Future research should address these systemic barriers, advocating for policy changes that promote mobility equity and inclusive environments for children with disabilities.

The results highlight the importance of close and continuous collaboration between professionals and families to ensure proper follow‐up, personalised adaptations and positive experiences. This study underscores the opportunity to continue researching and developing innovative solutions that enable greater inclusion and participation of children with motor limitations in various settings, both at home and in the community.

## 6 Conclusions

The implementation of early powered mobility interventions for children with SMA1 is powerful for fostering independence and improving participation for children and their families. The collected images and comments demonstrated that powered mobility not only facilitates movement but also promotes emotional well‐being and social engagement and enhances family interactions. However, addressing and overcoming architectural and accessibility barriers is essential to maximise the benefits of powered mobility.

## Author Contributions

**María Coello‐Villalón:** conceptualisation, investigation, methodology, writing – original draft, software. **Cristina I Díaz‐López:** investigation, formal analysis, methodology, writing – original draft, software. **Purificación López‐Muñoz:** conceptualisation, methodology, writing – original draft, investigation. **Helena Romay‐Barrero:** conceptualisation, investigation, writing – original draft, methodology. **Soraya Pacheco‐da‐Costa:** conceptualisation, investigation, writing – original draft, validation, software. **María Plasencia‐Robledo:** conceptualisation, investigation, writing – original draft, visualisation, formal analysis, data curation. **Egmar Longo:** conceptualisation, methodology, formal analysis, supervision. **Rocío Palomo‐Carrión:** funding acquisition, methodology, writing – review and editing, writing – original draft, supervision, project administration, resources, data curation.

## Conflicts of Interest

The authors declare no conflicts of interest.

## Data Availability

The data that support the findings of this study are available from the corresponding author upon reasonable request.
